# Potential biomarkers and their recent applications ofr point-of-care biosensors for the diagnosis of malaria

**DOI:** 10.1007/s00604-026-08026-2

**Published:** 2026-04-14

**Authors:** Leyla Karadurmus, Ahmet Cetinkaya, Pinar Kocabay, Menyar Ben Jaballah, Eva Baldrich, Sibel A. Ozkan

**Affiliations:** 1 Faculty of Pharmacy, Department of Analytical Chemistry, Adıyaman University, Adıyaman, Türkiye; 2 Gülhane Faculty of Pharmacy, Department of Analytical Chemistry, University of Health Sciences, Ankara, Türkiye; 3 Faculty of Pharmacy, Department of Analytical Chemistry, Ankara University, Ankara, Türkiye; 4 Graduate School of Health Sciences, Ankara University, Ankara, Türkiye; 5 Diagnostic Nanotools Group, Vall d’Hebron Hospital Institut de Recerca (VHIR), Barcelona, Spain; 6 Centro de Investigación Biomédica en Red de Enfermedades Infecciosas (CIBERINFEC), Instituto de Salud Carlos III, Madrid, Spain

**Keywords:** Malaria, Biosensors, Biomarkers, Point-of-care, Malaria diagnosis, Clinical diagnosis, Medical devices

## Abstract

Malaria remains to be one of the deadliest infectious illnesses and is especially common in underdeveloped and developing countries. Recent advances (2022–2025) in biosensor engineering, nanomaterials, and microfluidic automation have significantly expanded the capabilities of point-of-care diagnostic technologies for malaria. With the guidance of the World Health Organization (WHO) and the international community, malaria-endemic lands have achieved measurable advances in disease control and elimination. Globally, we are witnessing an increase in cases of clinically atypical malaria and asymptomatic carriers, which contributes to misdiagnosis and missed diagnosis. Other challenges include climate change, socioeconomic inequality, limited healthcare infrastructure, the emergence of new vector species, and increasing resistance to antimalarial drugs and insecticides. This shows that additional efforts will be needed if malaria is to be eradicated. Creating simple, practical, and affordable integrated biosensors is essential to diagnose early-stage malaria. In this context, one of the main challenges is converting complex biosensor techniques into affordable point-of-care testing (POCT) technologies that could be employed close to the patient without requiring skilled professionals or special infrastructures. Unlike recent reviews that primarily focus either on biomarker discovery or broadly on POC technologies, this work provides a comparative and mechanistic evaluation of electrochemical, optical, and piezoelectric biosensors, emphasizing primary research articles. Particular attention is given to signal transduction mechanisms, analytical trade-offs, microfluidic integration strategies, and real-world deployability considerations. This review gives general information about the biomarkers used for malaria diagnosis and summarizes some prominent examples of biosensors described in previous studies for malaria detection POCT making use of different transduction formats. The work finishes with some general conclusions, demanding to progress the design of portable point-of-care diagnostic technologies and further research to achieve sensitive quantification of biomarker panels as the key to the future of malaria detection and global control.

**Graphical Abstract:**

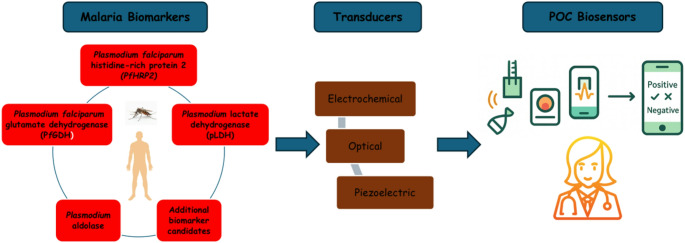

## Introduction

Malaria continues to be one of the deadliest infectious illnesses in the world, resulting in high rates of morbidity and mortality, particularly in tropical and subtropical areas. Public health systems have faced difficulties for centuries due to this disease, caused by *Plasmodium* parasites and spread by the bites of infected *Anopheles* mosquitoes. In a Malaria Ministerial Conference co-hosted by the WHO and the Government of Cameroon in March 2024, the ministers of health from the 11 high-burden to high-impact African countries signed the Yaoundé Declaration, stating that “no one should die from malaria given the tools and systems available”. Nonetheless, just in 2023, malaria had produced 263 million new infections and 597 000 deaths. At least 95% of these cases occurred in Africa, and over 70% occurred in children younger than 5 years. Despite significant efforts to prevent and eradicate malaria, each year, hundreds of millions of reported infections and hundreds of thousands of fatalities are declared [1, 2]. Since early and precise detection is essential to controlling and lessening the effects of the disease, there has never been a pressing need for more effective and efficient diagnostic techniques. Microscopy and rapid diagnostic tests (RDTs), the two most widely used malaria diagnostic techniques, have been essential to implement malaria control initiatives. Even if molecular methods provide multiplexed and more sensitive detection of the parasite, it is widely agreed that they face limitations for field implementation. Microscopy, which is regarded as the gold standard for diagnosing malaria, looks at dyed blood smears under a microscope to see whether *Plasmodium* parasites are present. Despite being extremely precise in the hands of an expert, this approach is not applicable in many resource-constrained environments because it necessitates specialized staff, a substantial amount of time, and laboratory facilities. Point-of-care (POC) refers to diagnostic testing performed near the patient, whereas point-of-care testing (POCT) describes the overall diagnostic system or procedure. POCTs are important because they can be performed close to the patient without complex technical skills or specialized equipment.

On the other hand, RDTs offer a speedier substitute by identifying specific *Plasmodium* antigens in a patient’s blood. However, most RDTs detect histidine-rich protein (HRP2), which is present only in *P. falciparum*. In addition, the sensitivity and specificity of RDTs vary between devices from different producers and between fabrication batches, and variables like parasite density and genetic changes in *Plasmodium* strains might impact performance [3]. The scientific community has made impressive progress in the last few years in finding new biomarkers for the diagnosis of malaria. Biomarkers are biological substances present in a patient’s sample that signal a certain illness condition and present the possibility of more quick, sensitive, and specific diagnostic techniques. These biomarkers constitute a major advancement in diagnostic capacities because they may be found in different types of biological samples [4]. Identifying particular biomarkers linked to the presence of the malaria parasite allows confirming a clinical malaria diagnosis, but may also disclose the parasite species and provide an estimate of the infection severity. Although *Plasmodium falciparum* histidine-rich protein 2 (PfHRP2) has been for long the reference biomarker to diagnose malaria, additional biomarkers are being targeted, such as *Plasmodium falciparum* glutamate dehydrogenase (PfGDH), *Plasmodium* lactate dehydrogenase (pLDH), *Plasmodium* aldolase, and hemozoin, in an attempt to detect more parasite species than just *P. falciparum* [4].

Biosensors are analytical tools that employ specific biological recognition elements to detect target analytes and have been widely applied in medical diagnostics [5]. Because biosensors are simple to use and selective to the target analyte, they can be employed for continuous monitoring of certain diseases and are compatible with assay multiplexing [6, 7]. Electrochemical biosensors are considered among the most important biosensors because they are easy to miniaturize and can be utilized for on-site diagnosis in affordable and portable biosensing devices [8, 9]. However, electrochemical detection on microchip-based technology has always depended on intricate detection amplification and has certain practical drawbacks, notably time consumption. Additionally, many microchip-based sensors require multiple washing steps and incubation with detection tags and/or reagents, complicating their use in POC settings [10]. Enzyme labels are most frequently employed in immunoassay with this aim [11], but immunosensors based on nanomaterial labels have recently been created [12, 13]. Many authors defend that such enzyme-free electrochemical sensors support fast and sensitive measurements. Nevertheless, sluggish electrode kinetics and poor signal stability are likely problems when using non-enzymatic electrochemical sensors [14]. So, enzyme-free and washing-free electrochemical immunosensors based on directed signal amplification strategies are required to detect the disease in an early stage in POC settings and satisfy the clinical demand [15]. Owing to their excellent sensitivity, wide linear detection range, robustness, and consistent reproducibility, electrochemical immunosensors have been widely used in malaria detection studies. Impedimetric techniques, which are appealing for high-sensitivity label-free detection, and label-based assays that combine colorimetry and amperometry are examples of detection methodologies. In addition, optical and piezoelectric biosensors have been used as distinct analytical methods for malaria detection [16].

However, global control of malaria requires more than just a rapid, accurate method for detecting cases. Only a sensitive test can identify asymptomatic carriers, who act as reservoirs for *Plasmodium* transmission and should be identified and treated to control and ultimately eradicate human malaria. Precise diagnosis, including parasite species identification, is needed to recommend personalized treatment, and quantitative detection over time may help study treatment response and anticipate drug resistance. One of the difficulties in diagnosing malaria is that the majority of the susceptible population resides in isolated areas with little access to medical care or where local medical facilities are inadequately equipped to offer optimal medical attention. Thus, cost-efficient diagnostic tests that do not require specialized equipment are needed. POCs are important in this situation because they can be performed close to the patient and do not require complex technical skills or specialized equipment. They enable rapid detection of analytes, accelerating disease monitoring, diagnosis, and treatment. Next-generation POCT will facilitate prompt medical decisions, as early diagnosis improves patient outcomes by enabling early treatment.

Several recent reviews have addressed malaria biomarkers and diagnostic technologies. For example, Harmonis et al. [17] focused primarily on biomarker discovery and early/asymptomatic detection, whereas Coro et al. [18] presented a broad scoping review of point-of-care technologies. However, a detailed comparative evaluation of biosensor transduction mechanisms, analytical performance trade-offs, microfluidic automation strategies, and recent primary technological developments remains limited. The present review aims to bridge this gap by integrating biomarker analysis with a critical comparison of electrochemical, optical, and piezoelectric biosensing architectures, emphasizing recent primary studies and highlighting practical implementation challenges.

## Biomarkers in the diagnosis of malaria

Malaria is diagnosed using standard techniques such as microscopic examination, immunochromatographic RDTs, and nucleic acid-based tests [19]. Some disadvantages of these approaches include their time-consuming nature, multi-step analyses, and limited practical applicability. Owing to these limitations, a malaria detection POC device that is easy to use, quick, affordable, sensitive, and on-site applicable is highly in demand. This can be accomplished by developing diagnostic sensors that detect one or more malaria biomarkers.

Biomarkers are naturally occurring chemicals, genes, or traits that can be used to identify a certain pathological, physiological, or disease process [20, 21]. Biomarkers are specific to a particular disease pathogen, making them useful for diagnostic tools [22]. Analytes such as PfHRP2, PfGDH, pLDH, *Plasmodium* aldolase, and hemozoin are among the biomarkers commonly employed in malaria-related detection equipment.

### *Plasmodium falciparum* histidine-rich protein 2 *(PfHRP2)*

PfHRP2 is the biomarker most widely targeted by currently available diagnostic immunoassays for detecting malaria caused by Plasmodium falciparum, which produces PfHRP2 abundantly and continuously throughout its life cycle. Histidine makes up to 37% of the amino acid sequence of PfHRP2, and 85% of its sequence is made up of a repeat of histidine plus alanine [23]. The parasites multiply within host Plasmodium-infected red blood cells (RBCs), releasing large amounts of the water-soluble PfHRP2 protein into their cytoplasm. PfHRP2 also accumulates on the surface of the membrane of the *Plasmodium*-infected RBCs, the food vacuoles, the digestive vacuoles, as well as in the patients’ urine, serum, plasma, and cerebrospinal fluid. Due to its abundance relative to other biomarkers, PfHRP2 is widely used as a target for the development of antimalarial medications and as a preferred target in rapid diagnostic tests. Nevertheless, malaria diagnosis using PfHRP2 is influenced by parasite density, antigen production levels, and the antigen’s persistence in the bloodstream and detection is impaired by the growing prevalence of deletions in the PfHRP2 gene in some geographic areas. In addition, PfHRP2 is exclusive of *P. falciparum*, and RDTs based on detection of this biomarker do not detect the other human-infecting *Plasmodium* species.

### *Plasmodium* lactate dehydrogenase (pLDH)

All human-infecting Plasmodium species contain the common biomarker pLDH. By employing a cofactor-reduced NADH, pLDH primarily converts pyruvate to lactate [22]. The asexual intraerythrocytic cycle produces high levels of pLDH. Because pLDH differs structurally from human lactate dehydrogenase (hLDH), it can serve as a malaria biomarker. Some authors argue that pLDH can be used in malaria case management because, within 24 h of successful treatment, the enzyme’s circulation in the host environment ceases. Consequently, detection of pLDH should yield fewer false-positive diagnoses after infection suppression than detection of more persistent biomarkers, such as PfHRP2 [24]. In contrast, pLDH detection can result in more false negatives in patients displaying low parasitemia than detection of PfHRP2 because compared to PfHRP2, pLDH exhibits reduced expression levels.

### *Plasmodium falciparum* glutamate dehydrogenase (PfGDH)

Glutamate dehydrogenases (GDHs) are widely distributed enzymes that link nitrogen and carbon metabolism. *P. falciparum* produces PfGDH during its intra-erythrocytic cycle. Interestingly, PfGDH differs from the host’s GDH with respect to kinetics, structural features, location within the cell, and sequence, which allows exploiting PfGDH as a biomarker of malaria infection. PfGDH proteins are encoded by three distinct genes expressed by *P. falciparum*. Chromosome 14 contains gdhA and gdhB, while chromosome 8 contains gdhC.

### *Plasmodium* aldolase

The protein aldolase is a homotetrameric enzyme involved in glycolysis, with each subunit weighing about 40 kDa. Aldolase is expressed by all human-infecting *Plasmodium* species, making it a pan-specific antigen for detecting *Plasmodium* infections. While human aldolase and *Plasmodium* aldolase differ in sequence, *P. falciparum* and *P. vivax* display a 369 amino acid conserved enzyme [25]. The parasite’s cytoplasm contains aldolase in its soluble and active form. Here, in addition to its role as a glycolytic enzyme, Plasmodium aldolase is known to bind to actin and act as a bridge to protein receptors on the parasite’s membrane, which seems to play a crucial role in parasite protein reassembly and host cell entry. Although many authors have proposed aldolase as a promising biomarker for pan-*Plasmodium* infections, it is far from displacing PfHRP2 in commercially available RDTs. The main reason the low concentration of aldolase produced by Plasmodium-infected RBCs [26]. In addition, *Plasmodium falciparum* aldolase shares 61–68% sequence identity with eukaryotic aldolases, another drawback for its use as a biomarker of malaria infection.

### Additional biomarker candidates for malaria diagnosis and assessment

In addition to the enzymes detailed above, a growing number of *Plasmodium* enzymes are being explored as biomarker candidates for pan-malaria diagnosis [27]. Some examples are phosphoethanolamine N-methyltransferase (PMT), that is participated in parasite lipid biogenesis and gametocyte development and is expressed throughout the intra-erythrocytic cycle; thioredoxin peroxidase 1 (TPx-1), a parasite housekeeping enzyme located in its cytoplasm that plays a role in mitigating oxidative stress; heme detoxification protein (HDP), that is a thermostable enzyme unique to *Plasmodium* that is in charge of convert toxic heme into hemozoin while feeding on the host RBCs [27].

Hemozoin is an insoluble brown microcrystalline substance produced during blood digestion by blood-feeding parasite species like *Plasmodium falciparum* [20, 22]. Its crystal structure comprises hemes connected by the core ferric ion and the carboxylate side group oxygen. Amino acids and toxic-free heme produced during hemoglobin digestion are polymerized to hemozoin during the intra-erythrocytic cycle, when *Plasmodium* infects the host’s RBCs. In addition, the parasite breaks down 80% of the hemoglobin in infected RBCs [28, 29]. Hemozoin exhibits unique magnetic, optical, and acoustic properties, prompting researchers to explore it as a target in a range of novel label-free diagnostic methods [30].

*Anopheles* mosquitoes deliver saliva into the host’s bloodstream while feeding on blood and this saliva contains a mixture of mosquito molecules (proteins, enzymes) that can elicit immune responses in humans. It has been proposed that measuring in the population these antibodies against mosquito saliva components can help detect recent exposure to mosquito bites [31].

Apart from biomarkers for malaria diagnosis, several parameters can assess the severity of the infection, which is important for guiding treatment selection and monitoring the patient’s response to therapy. For instance, *Plasmodium* parasites invade the host RBCs, leading to their destruction. Accordingly, severe malaria often results in anemia, which may even require blood transfusions. Severe malaria is also associated with thrombocytopenia, which may disrupt normal hemostasis and increase the risk of hemorrhage. In addition, changes in coagulation indicators such as D-dimer and fibrinogen can signal a predisposition to coagulopathy and thromboembolic complications. Elevated inflammatory mediators, including tumor necrosis factor (TNF) and interleukin-6 (IL-6), further contribute to disease severity and are implicated in complications such as cerebral malaria.

## Biosensors as POCT tools

Biosensor development is a dynamic, rapidly evolving field forged by the interaction of diverse disciplines. This makes it possible to design biosensors as cost-efficient, portable technologies that deliver swift, reliable results, with simple handling and interpretation, even for non-specialist personnel, which makes them ideal tools for POCT [5]. Furthermore, compared to classical RDTs, which typically provide qualitative outputs, most biosensors quantify their targeted biological or chemical analytes with high specificity and sensitivity, allowing them to compete with more expensive, bulkier, and sophisticated laboratory equipment. The measurement mechanism of a biosensor begins with the interaction between the transducer-bound bioreceptor and the target analyte. This interaction generates a response that the transducer converts into an electrical signal. This signal is next processed and converted into a measurable parameter, which is then displayed on the user’s interface [32, 33]. This basic operating mechanism may vary depending on the transducer and bioreceptor types, but the fundamental principle remains the same. Among the various transduction methods that have been utilized for developing biosensors, electrochemistry is the most widely adopted one for its high sensitivity, cost-effectiveness, and simplicity [34].

POCT is a medical diagnostic procedure performed near or at the patient’s site. This eliminates the need for sample transfer to a laboratory or for technician assistance, simplifying the diagnostic process. Therefore, POCT enables patients to get rapid results, decreases the number of hospital visits, and lowers the cost of the procedure. Ideally, they could also allow healthcare professionals to track patients’ status and intervene remotely in a timely manner in emergencies. In this context, it is generally agreed that a POCT device should be affordable, easy to use, time-saving, and usually hand-held or at least portable.

Different technologies have been exploited to develop POCT devices [35–37]. For instance, wearable biosensors, integrated into devices such as smart shirts, wristbands, or contact lenses, have been produced to continuously monitor vital signs like blood sugar levels, blood pressure, and heart rate, providing real-time health data [38]. However, detecting infection biomarkers in clinical samples often requires more sophisticated sample preprocessing and multi-step assay automation, which, in turn, demands more complex device architectures. Microfluidic-based POC platforms have been extensively employed for this purpose [39, 40].

Microfluidic integration plays a critical role in improving biosensor robustness, particularly for electrochemical systems prone to surface fouling and non-specific adsorption when analyzing whole blood. Recent microfluidic platforms incorporate passive plasma separation, magnetic bead manipulation, automated reagent delivery, and integrated washing steps, thereby reducing manual intervention and enhancing reproducibility. Microfluidic biosensors integrate microfluidic features, such as microchannels and cells, to achieve the miniaturization and automation of analytical processes. In this way, reactions occur in minute volumes and at fast rates. This contributes to reducing the volume of samples and reagents needed, minimizing waste generation, and providing more sensitive analyte detection in extremely short assay times. In contrast, microfluidic devices usually depend on the precise control of liquid flow, for which integration of external (micro)pumps may be required. Microfluidic channels can also be obstructed by particulate matter in the samples and by bubbles, demanding previous sample preprocessing and slow flow rates, which may complicate the analysis of complex biological samples.

A few examples have been reported employing microfluidic devices for the detection of malaria biomarkers. For instance, the research by Xiu et al. [41] presents an equipment-free microfluidic platform for malaria screening and genotyping of the *Plasmodium* species. The platform enhances diagnostic accuracy and efficiency by integrating recombinase polymerase amplification (RPA), clustered regularly interspaced short palindromic repeats (CRISPR), and microfluidics. The authors defend that the high sensitivity (98.41%) and specificity (92.86%) of this biosensor make it a practical solution for resource-limited settings where access to advanced diagnostic facilities is limited. Their microfluidic cartridge integrates recombinase polymerase amplification (RPA) and CRISPR modules within compartmentalized microchannels, enabling automated nucleic acid amplification and detection without external laboratory infrastructure. Fluid routing ensures sequential reagent handling and minimizes contamination risk.

**Fig. 1:**
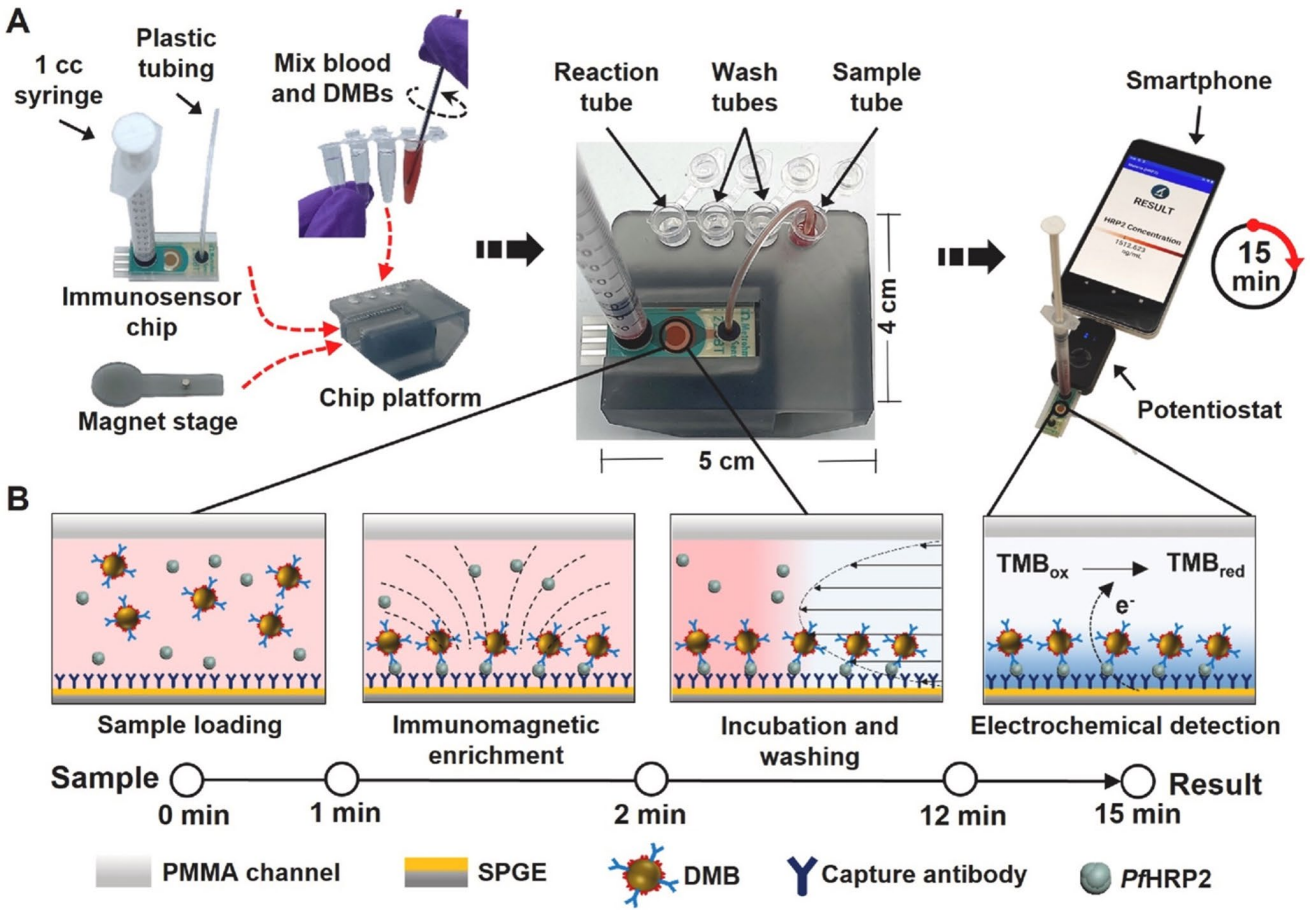
Design and operational mechanism of the microfluidic immunoassay described in [42] for the rapid detection of malaria. Reprinted from Ref. [42] with permission from Elsevier

In another study, Li et al. [42] produced a microfluidic immunoassay for the rapid determination and prognosis of malaria infection by quantifying PfHRP2 (Fig. 1). The device utilizes a microfluidic chip, a compact platform capable of manipulating very small volumes of samples and detecting low biomarker concentrations. This enables early diagnosis of malaria even before the onset of symptoms. Beyond its diagnostic use, the device can monitor disease progression and treatment effectiveness by tracking PfHRP2 levels over time, contributing to improved healthcare services in endemic regions. As with many POCT devices, it is designed to be portable, offering quick results with only 10 µL of blood sample and requiring minimal manipulation. The microfluidic immunoassay employs capillary-driven flow to automate incubation and washing steps within a compact chip architecture. Integration of the electrochemical detection chamber within the microfluidic layout enhances signal stability while reducing matrix interference from complex biological samples.

These examples illustrate how the sensor’s low detection limits can combine with the ability to operate without additional laboratory equipment or power sources provided by microfluidic platforms, giving shape to valuable advancements in the expanding field of POC biosensors. The next sections will discuss some examples of biosensors reported for the diagnosis of malaria, a field where rapid and accurate disease detection is essential.

## Biosensors for malaria POCT

Malaria is a significant economic and health burden worldwide, with approximately 600,000 deaths and 263 million cases in 2023 [2]. While efficient medicines are available that, when used promptly, can significantly lessen disease severity, malaria, especially severe *Plasmodium falciparum* infections, is a cause of death and morbidity even in non-endemic areas. Even though multiple studies have demonstrated that it is not a reliable indicator of severity, especially in non-endemic locations, hyperparasitemia remains the primary criterion to diagnose severe malaria. The gold standard method used to diagnose malaria and determine hyperparasitemia is microscopy, which needs trained staff [43]. Molecular methods such as PCR are positioned as competitive alternatives when centralized laboratories are available, but rely on well-equipped infrastructures incompatible with POCT. Recent research has highlighted the potential of immunochromatographic RDTs for malaria POCT, emphasizing their popularity and effectiveness in providing rapid results. However, RDTs fail to identify low parasitemias, also known as submicroscopic malaria, and provide only qualitative results that depend on subjective interpretation [44, 45]. In response, the World Health Organization fostered an initiative to eradicate malaria, with one of its key objectives being the development of cost-efficient rapid tests for diagnosing the disease. Achieving this goal will require the joint work and efforts of researchers and healthcare professionals, but also political, regulatory, and funding agents.

Biosensors have appeared as viable options for fast and reliable determination of malaria [5, 46–48]. Finding sensitive and specific biomarkers will be critical to developing dependable diagnostic tools to support tailored treatment for patients who test positive for malaria. Biosensors targeting a variety of biomarkers associated with the disease have been reported to provide fast and effective testing in clinical and POC environments. Progress in biosensor development for malaria diagnosis aligns with the broader trend of integrating advanced technology into the medical field. By leveraging biosensor capabilities, researchers hope to revolutionize the diagnosis and surveillance of malaria, enabling early disease detection, facilitating the implementation and control of therapeutic interventions, and contributing to worldwide efforts to eradicate malaria [47–50]. A comparative summary of analytical performance, sample requirements, and response times of recently reported biosensors is provided in Table 1 to facilitate cross-platform evaluation.

**Table 1:** Classification and performance characteristics of biosensors reported for detecting malaria biomarkers

Biomarker	Sensing Technique	LOD	Range	Sample	Response Time	Ref.
PfLDH	Amperometric	200 ng/mL	-	whole blood samples	< 20 min	[51]
PfLDH	Amperometric	1–3 ng/mL	3 and 25 ng/mL	diluted blood samples	25 min	[52]
pLDH	Chronoamperometric	0.019 ng/mL and 0.023 ng/mL	-	buffer and serum samples	< 2 h	[53]
PfHRP2	Chronoamperometric	36 pg/mL buffers 40 pg/mL samples	-	spiked serum samples	-	[54]
PfHRP2	CV	N.A	250 pg/mL-100 ng/mL	rabbit and human blood serum samples	30 ± 5 min	[55]
PfLDH	DPV	0.5 fM	-	real samples	-	[56]
PfHRP2	DPV	2.8 ng/mL	10–500 ng/mL	human serum samples	45 min	[57]
	EIS	3.3 ng/mL	10–400 ng/mL			
*P. vivax* MSP1 antigen	EIS	~ 40 Pv-infected RBCs	-	blood sample	5 min	[58]
PfGDH	EIS	0.77 pM	100 fM to 100 nM	human blood serum	-	[59]
PfLDH	EIS	1.49 pM	4.5 pM–100 nM	diluted and whole serum	7 h	[60]
PfGDH	Electrochemical/FET	48.6 pM	100 fM – 10 nM	Serum	5 s	[61]
β-hematin	SWV	0.43 µg/mL	1.96–9.91 µM	human blood serum	-	[62]
PfHRP2	SWV	3.73 nM	0–250 nM	serum buffers	5–6 min	[63]
PfLDH	SWV	N.A	0–700 nM	diluted human blood	-	[64]
PfLDH	Fluorescence	0.92 ng/mL	3–153 ng mL^− 1^	human blood	20 min	[65]
PfLDH	Fluorescence	0.90 ng mL^− 1^	0.78–12.5 ng mL^− 1^	human blood	15 min	[66]
PfLDH	Fluorescence	10 amole	-	buffer	-	[67]
PfLDH	Immuno‑PCR	0.02 parasite/µL	-	-	-	[68]
*Plasmodium*-infected RBCs	Optical refractive-index sensing	0.0007 refractive index units (RIU)	-	clinical samples	-	[69]
PfGDH	Optical	264 pM	-	buffer	-	[70]
*Plasmodium*-infected RBCs	Surface Plasmon Resonance	Ring 1.396, trophozoite 1.381, and schizont stages 1.371 (RIU)	1.369–1.409 (RIU)	-	-	[71]
*Plasmodium*-infected RBCs	Surface Plasmon Resonance	Ring 142.857 μm/RIU, Trophozoite 123.684 μm/RIU and Schizont stages 120.687 μm/RIU	-	-	-	[72]
*Plasmodium*-infected RBCs	Surface Plasmon Resonance	1.29 × 10^− 5^ RIU schizont, 1.09 × 10^− 5^ RIU trophozoite, 8.04 × 10^− 6^ RIU ring phases	-	-	-	[73]
*ffPlasmodium*-infected RBCs	Surface Plasmon Resonance	1.370–1.410 RIU	-	Blood/red blood cells	-	[74]
Antigen–antibody binding	Piezoelectric	3.43 × 10^− 4^	-	Theoretical modeling	-	[16]

### Electrochemical biosensors

Electrochemical biosensors operate by converting biochemical interactions at an electrode interface into measurable electrical signals. In amperometric and chronoamperometric systems, redox reactions generate current proportional to analyte concentration. Voltammetric techniques such as cyclic voltammetry (CV) and differential pulse voltammetry (DPV) measure current–potential profiles resulting from oxidation–reduction processes. In label-free electrochemical impedance spectroscopy (EIS), biomarker binding alters charge transfer resistance and double-layer capacitance, enabling quantitative detection without enzymatic labels. Electrochemical biosensors have proven to be the most promising technology for POCT, with an edge in terms of speed of result generation, ease of use, cost-effectiveness, and in situ testing capabilities [34]. Comparatively, amperometric and labeled voltammetric systems often achieve lower detection limits and shorter response times but require additional reagents and incubation steps. Label-free impedance systems reduce reagent complexity but may be more susceptible to matrix effects and electrode fouling. Aptamer-based sensors provide enhanced thermal stability and lower production costs compared to antibody-based immunosensors; however, surface chemistry optimization and blocking strategies remain critical for reproducibility in complex samples. Notable examples have been described for diagnosing various infectious diseases, including malaria, with a strong focus placed on rapid and specific diagnostics [5, 44–48, 75–80]. This technology represents a convenient tool for POC analysis, with quick responsiveness, potential for miniaturization, and ease of use. By leveraging the advancements in nanotechnology, materials science, and biorecognition elements, these biosensors are poised to play a crucial role in allowing early diagnosis, ongoing monitoring, and effective management of health conditions at the point of care.

A few electrochemical biosensors have been reported in the literature for diagnosing malaria [19, 77]. These biosensors predominantly detect biomarkers, such as specific proteins or genetic sequences of *Plasmodium*, associated with the malaria parasite in patient blood samples. By analyzing the electrochemical signals produced through the association between the target biomarker and the sensor, such tools can effectively determine the presence of the parasite. Overall, using electrochemical biosensors for diagnosing malaria holds strong potential for increased testing speed and accuracy, particularly in settings lacking access to traditional testing methodologies. This section will discuss the underpinnings, applications, and current trends for electrochemical POC biosensors in diagnosing malaria.

**Fig. 2:**
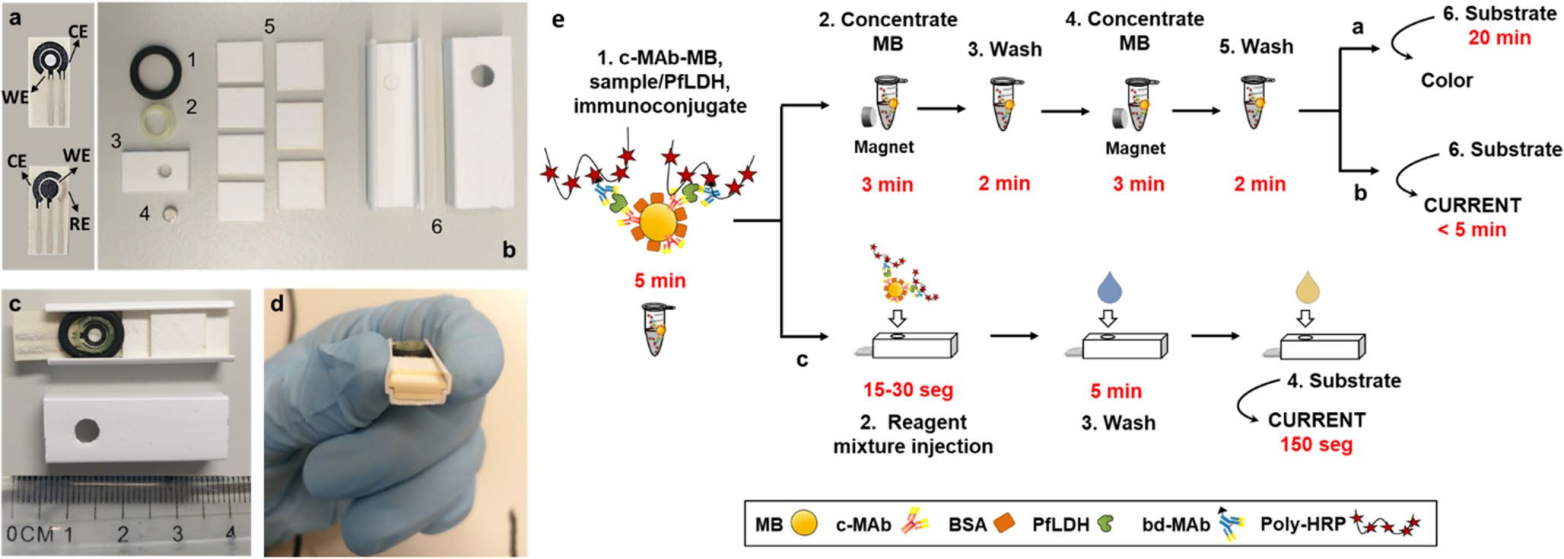
Disposable paper electrode microfluidic for the determination of PfLDH. (**a**) Double-sided paper SPCE. (**b**) Key components of the device. (**c**-**d**) The assembled device, showing the configuration of the absorbent pad stack. e) Scheme comparing the level of handling and assay time for the classical assay carried out in tubes and the paper-based alternative proposed. Reprinted from Ref. [51] with permission from Elsevier

For instance, Ruiz-Vega et al. developed a rapid electrochemical POC device for detecting *Plasmodium falciparum* LDH (PfLDH) in whole blood using low-cost materials (Fig. 2) [51]. The method includes 5-minute cell lysis to release parasites, followed by stirring with immunomodified magnetic beads, antibody detection, and signal amplification. The rest of the assay and the electrochemical detection occur on a disposable paper electrode microfluidic device. The sensor detects PfLDH at levels as low as 2.47 ng/mL and parasitemia between 0.006% and 1.5%, distinguishing healthy individuals from malaria patients with parasitemia above 0.3%. This device offers fast, quantitative malaria diagnosis with minimal user intervention.

**Fig. 3:**
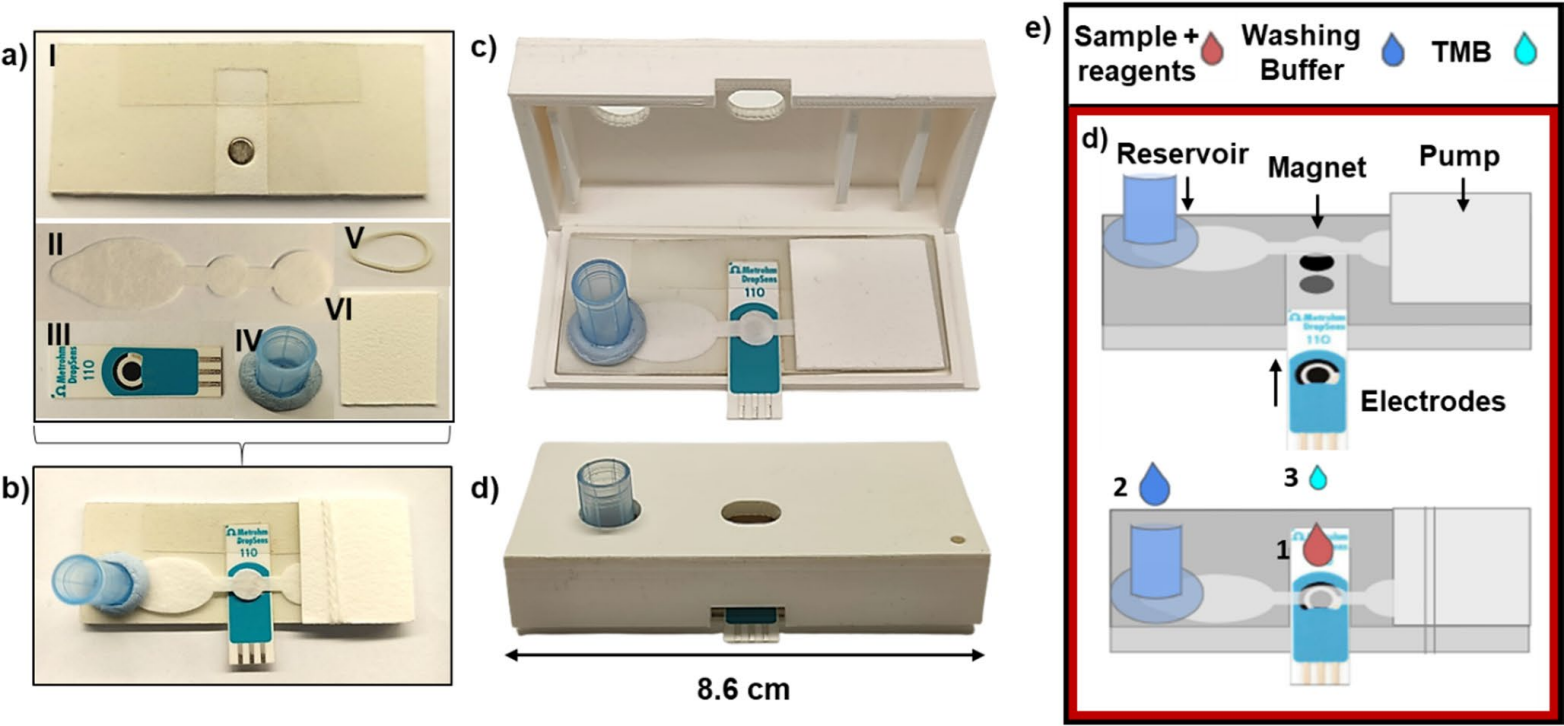
MED components and assembly. Reprinted from Ref. [52] with permission from Elsevier

In a subsequent study, the same team developed low-cost, easy-to-use paper-based microfluidic electroanalytical devices (PMEDs) (Fig. 3) [52]. The authors showed that PMEDs could detect different magneto-assays targeting analytes such as streptavidin-conjugated horseradish peroxidase (HRP), biotinylated HRP, and PfLDH in 25 min. Furthermore, measurements could be carried out using either commercial or customized SPCE and measurement apparatus, demonstrating the versatility of the reported technology. In diluted blood samples, PfLDH detection showed a linear response over 3–25 ng/mL, with a detection limit of 1–3 ng/mL and a quantification limit of 6–12 ng/mL. This technology is an ideal solution for POC testing because it is cost-effective and simple.

Hemben et al. successfully developed an immunosensor to detect pan malaria pLDH antigen and compared its performance to that of OptiMAL-IT and BinaxNow Malaria commercial kits [53]. The sensor was finely-tuned for optimal sensitivity before its application in pLDH detection in serum samples. The developed sensor detected pLDH in buffer and serum samples with limits of detection (LODs) of 1.80 ng/mL and 0.70 ng/mL, respectively. The sensor’s sensitivity was further increased with AuNP coupled to the detecting anti-pLDH antibody, yielding LODs of 19 pg/mL in buffer and 23 pg/mL in serum samples.

**Fig. 4:**
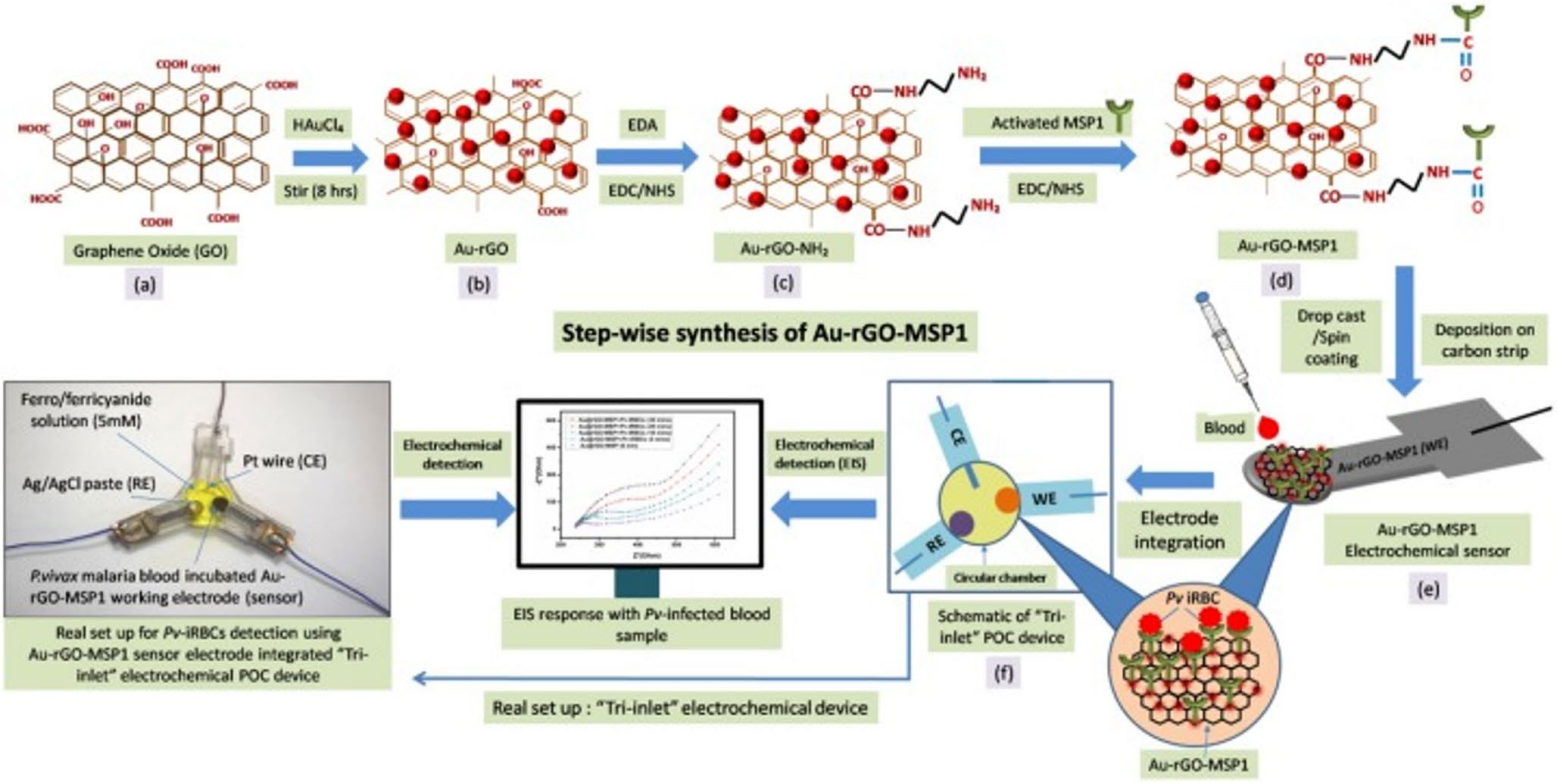
Schematic representation of the electrochemical “Tri-inlet” device integrated with the Au-rGO-MSP1 electrode. Reprinted from Ref. [58] with permission from Elsevier

In recent research, a novel POC electrochemical device with a “Tri-inlet” design has been designed for the early diagnosis of *Plasmodium vivax* (Pv) malaria (Fig. 4) [58]. The device’s core is an electrode sensor fabricated through a multi-step process. This includes synthesizing rGO-based gold nanocomposite (Au-rGO) and conjugating it with an antibody targeting the *P. vivax* MSP1 antigen. This antibody-nanocomposite complex (Au-rGO-MSP1) is then deposited onto a carbon strip. The sensor is engineered for high sensitivity by using ethylenediamine (EDA) as a spacer to ensure the optimal orientation of the antibody on the electrode surface. Testing with whole blood samples from patients demonstrated the device’s high sensitivity and selectivity, with an LOD as low as ~ 40 Pv-infected RBCs per 10 µL of blood in just 5 min. This performance is on par with or superior to existing commercial malaria detection kits. The study suggests this electrochemical sensor could be turned into a disposable, on-site diagnostic device for early determination of *Plasmodium vivax* malaria.

Obisesan et al. reported the fabrication of electrochemical nanosensors using synthesized MO nanoparticles deposited on a gold electrode [62]. These nanosensors were specifically designed to detect the malaria biomarker β-hematin. The performance of the developed sensors was evaluated, and it was found that their current response to β-hematin was significantly higher than that of a bare gold electrode. Among the various configurations tested, the order of effectiveness was determined as Au-CuO (C) > Au-CuO (M) > Au-Fe₂O₃ (M) > Au-Fe₂O₃ (C) > Au-Al₂O₃ (M) > Au-Al₂O₃ (C) > bare Au. The sensors demonstrated stability with a relatively low current drop (10.61–17.35%) in the presence of the analyte. Notably, the Au-CuO sensor exhibited the best performance, effectively detecting *Plasmodium berghei* in serum samples from infected mice at concentrations of 3.60–4.8 mM and *Plasmodium falciparum* in human blood serum at concentrations of 0.65–1.35 mM.

Dip Gandarilla et al. developed a new one-step enzyme-free dual electrochemical immunosensor for detecting PfHRP2 [57]. The sensor was constructed by functionalizing a gold electrode with anti-PfHRP2 antibodies (Ab-PfHRP2), using a di hexadecyl phosphate (DHP) polymer as the immobilization platform. Different characterization techniques, such as CV, EIS, Fourier transform infrared spectroscopy (FTIR), scanning electron microscopy (SEM), and atomic force microscopy (AFM), were then used to analyze the sensor’s functional performance and structural integrity. The immunosensor was used for indirect PfHRP2 detection via DPV and EIS, showing a linear detection range of 10–400 ng/mL and 10–500 ng/mL for EIS and DPV, respectively. The LOD was 3.3 ng/mL for EIS and 2.8 ng/mL for DPV. When tested with human serum samples, the sensor’s performance was comparable to that of an ELISA test. Additionally, intra- and inter-assay variability were below 5%, suggesting that the immunosensor was a reliable, straightforward tool for in situ malaria biomarker detection.

Another study reported a sensitive and low-cost electrochemical immunosensor for detecting PfHRP2 [54]. Initially, an ELISA was used to assess the effectiveness of the immunoreagents for potential sensor applications. An Au sensor incorporating both a counter electrode and an Ag/AgCl reference electrode was selected and analyzed prior to the development of the immunosensor. Anti-PfHRP2 monoclonal antibodies were immobilized on the sensor as capture receptors, and a sandwich ELISA configuration was employed, with HRP as the enzyme reporter, generating an electrochemical response in the presence of a tetramethylbenzidine (TMB)/H₂O₂ system. The sensor displayed an LOD of 2.14 ng/mL for spiked buffers and 2.95 ng/mL for spiked serum. The use of AuNPs conjugated with antibody-linked enzymes enhanced signal amplification, resulting in improved detection limits of 36 pg/mL in buffer and 40 pg/mL in serum samples. These results suggested that this specific sensor configuration was well-suited for POC diagnostics for use in controlling and eradicating malaria in low-resource settings.

**Fig. 5:**
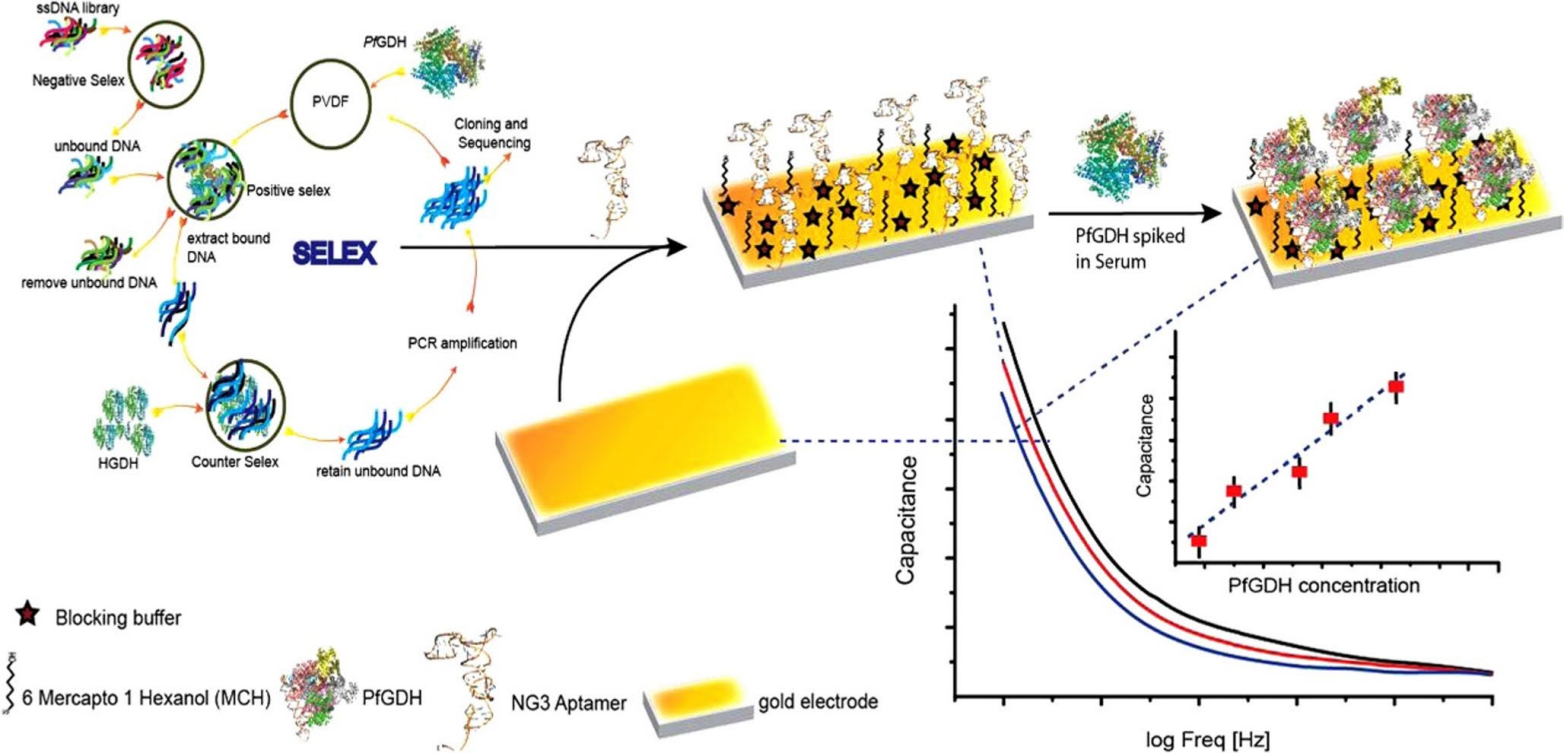
Selection of a PfGDH-binding aptamer through SELEX and fabrication of an aptasensor [59]. Reprinted from Ref. [59] with permission from Elsevier

Unlike conventional RDTs, which are primarily antibody-based and can be expensive and less robust, a number of teams have employed as an alternative aptamer, which are synthetic receptors selected in vitro through a process of SELEX. Singh et al. designed a capacitive aptasensor to detect PfGDH in human serum (Fig. 5) [59]. The sensor employs a thiolated single-stranded DNA (ssDNA) aptamer (NG3) that binds specifically to PfGDH with high affinity (Kd = 79 nM). The aptasensor operates on a non-Faradaic EIS platform, producing a capacitance response at an optimized frequency of 2 Hz. The sensor demonstrated a broad dynamic range from 100 fM to 100 nM, with an LOD of 0.77 pM in human serum. Importantly, it showed negligible interference from other malarial biomarkers such as PfLDH and PfHRP2. This label-free, highly sensitive aptasensor holds excellent potential for diagnosing asymptomatic malaria and monitoring disease regression during antimalarial treatment.

Another study focused on improving aptamer-based detection of PfLDH in complex matrices like human serum by optimizing the use of polyethylene glycol (PEG) blocking molecules to reduce non-specific adsorption [60]. EIS measurements and AFM, X-ray photoelectron spectroscopy (XPS), and quartz crystal microbalance with dissipation monitoring (QCM-D) analyses revealed that while PEG density remained constant, the receptor layer’s morphology changed significantly with incubation time. Shorter incubation times enhanced sensor performance by better exposing aptamer molecules, but prolonged incubation led to decreased sensor signals due to phase separation between aptamers and PEG. This phase separation increased aptamer density locally but hindered binding to PfLDH. Compared to hydrophobic blockers such as 6-mercapto-1-hexanol (MCH), PEG improved matrix tolerance, dynamic range, and detection limits, achieving optimal performance after 7 h of PEG incubation. The system proved effective in diluted and whole serum.

Using graphene oxide (GO) as an immobilization matrix, Jain et al. studied the ssDNA aptamer P38, which had been enriched against PfLDH through SELEX [56]. GO-P38 complexes were deposited on a glassy carbon electrode (GCE), and the modified electrode was then examined for PfLDH detection. The ID/IG intensity ratios in the Raman spectra, 0.67, 0.915, and 1.35 for graphite, GO, and P38-GO, respectively, confirmed that P38 was immobilized on GO. The electrode surface height increased from around 3.5 nm for GO-GCE to about 27 nm for P38-GO-GCE in the AFM investigation, indicating the creation of the P38 layer over GO-GCE. The designed aptasensor was shown to detect as low as 0.5 fM of PfLDH upon target challenge.

Lo et al. presented a new electrochemical aptamer-based molecular beacon biosensor for detecting *P. falciparum*, the deadliest malaria species, using aptamers specific for PfHRP2 [63]. The aptamers were modified with a methylene blue reporter and attached to an Au sensor surface for SWV measurements. Subsequent binding of PfHRP2 induced conformational changes in the aptamer, which changed the distance between the electrode and the tag and thus peak height. The developed biosensor demonstrated the ability to detect PfHRP2 in human serum with an LOD of 3.73 nM. Furthermore, the sensor was stable in serum buffers and reusable for multiple detection cycles, making it a cost-effective and reliable alternative for malaria diagnosis.

**Fig. 6:**
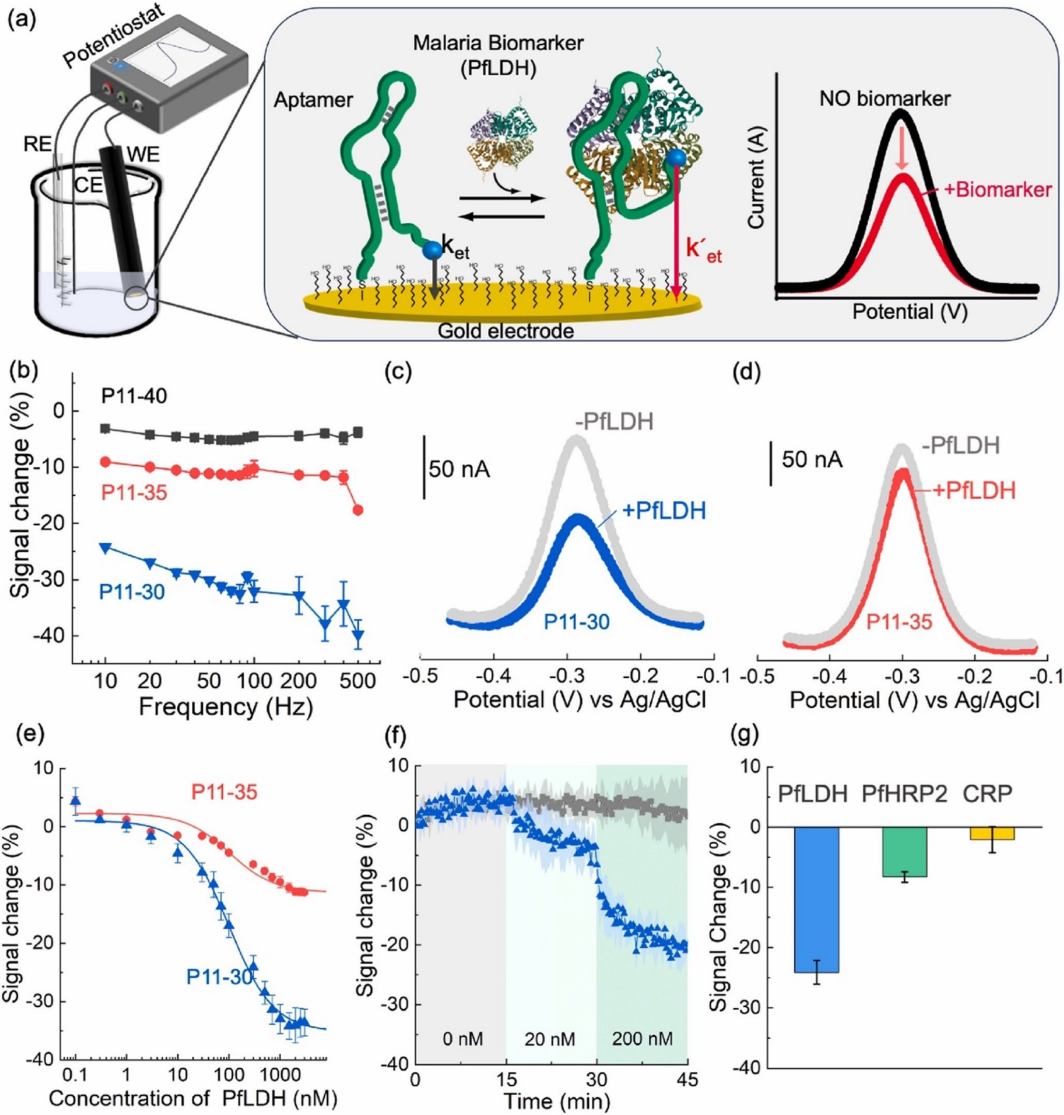
The results of electrochemical optimization and characterization of electrochemical molecular beacon aptasensor. Reproduced from Elsevier (Open access).(Access date: 9 January, 2025 [64]

Another study reported the fabrication and characterization of a similar electrochemical molecular-beacon aptasensor for convenient, rapid measurement of PfLDH (Fig. 6) [64]. Three sequences, P11-40, P11-35, and P11-30, were determined to be the best choices after they gradually narrowed down the list of the most promising aptamer candidates using optical characterization. They then proved they could create responsive aptasensors by testing them on an electrochemical platform. P11-30 was chosen for its superior analytical performance, demonstrating the ability to quickly and accurately identify the target at nM levels. Lastly, they demonstrated how this aptasensor can detect PfLDH in diluted human blood, offering a new method of diagnosing malaria.

### Optical biosensors

Optical biosensors detect variations in specific optical characteristics, such as absorption, fluorescence, or refractive index at the transducer’s surface, produced when a target analyte comes in contact with a respective bioreceptor [19, 79–81]. Optical platforms such as surface plasmon resonance (SPR) offer highly sensitive and real-time label-free detection. However, many SPR configurations rely on precise optical alignment and external instrumentation, potentially limiting portability in low-resource environments. Paper-based fluorescence and colorimetric systems provide improved field applicability but may exhibit reduced quantitative precision compared to laboratory-based optical setups. Thus, a trade-off exists between analytical sensitivity and operational simplicity. Accordingly, detection is carried out using light and can be accomplished with either label-based or label-free techniques. In label-based biosensors (usually using colorimetric, fluorescent, or luminescent tags), the intensity of the color, fluorescence, or light registered is proportional to the analyte concentration in the sample. Examples include colorimetric biosensors, microarrays, and many optic fiber biosensors. However, some optical sensors are also susceptible to changes produced in the surrounding medium and can be used for real-time detection of refractive index change on the transducer-medium dielectric interface. This allows for the easy detection of binding events or the measurement of analyte concentration in a label-free format. Well-known examples are the biosensors based on interferometers, optical waveguides, surface plasmon resonance (SPR), localized surface plasmon resonance (LSPR), and surface-enhanced Raman spectroscopy (SERS) [19, 82].

Guirgis reported a homogeneous fluorescent immunoassay for detecting *Plasmodium falciparum* heat shock protein 70 (Pf Hsp70) in infected blood cultures [83]. The assay used a recombinant PfHsp70 tagged with a fluorescent label, which became quenched upon interaction with AuNPs coated with anti-PfHsp70 monoclonal antibodies. When incubated with samples, any fresh antigen present displaced the fluorescent competitor from the AuNPs, which resulted in an increase of fluorescence intensity. The assay demonstrated a 2.4 µg/mL LOD and could detect PfHsp70 in Malaria-positive human blood cultures with 3% parasitemia.

**Fig. 7:**
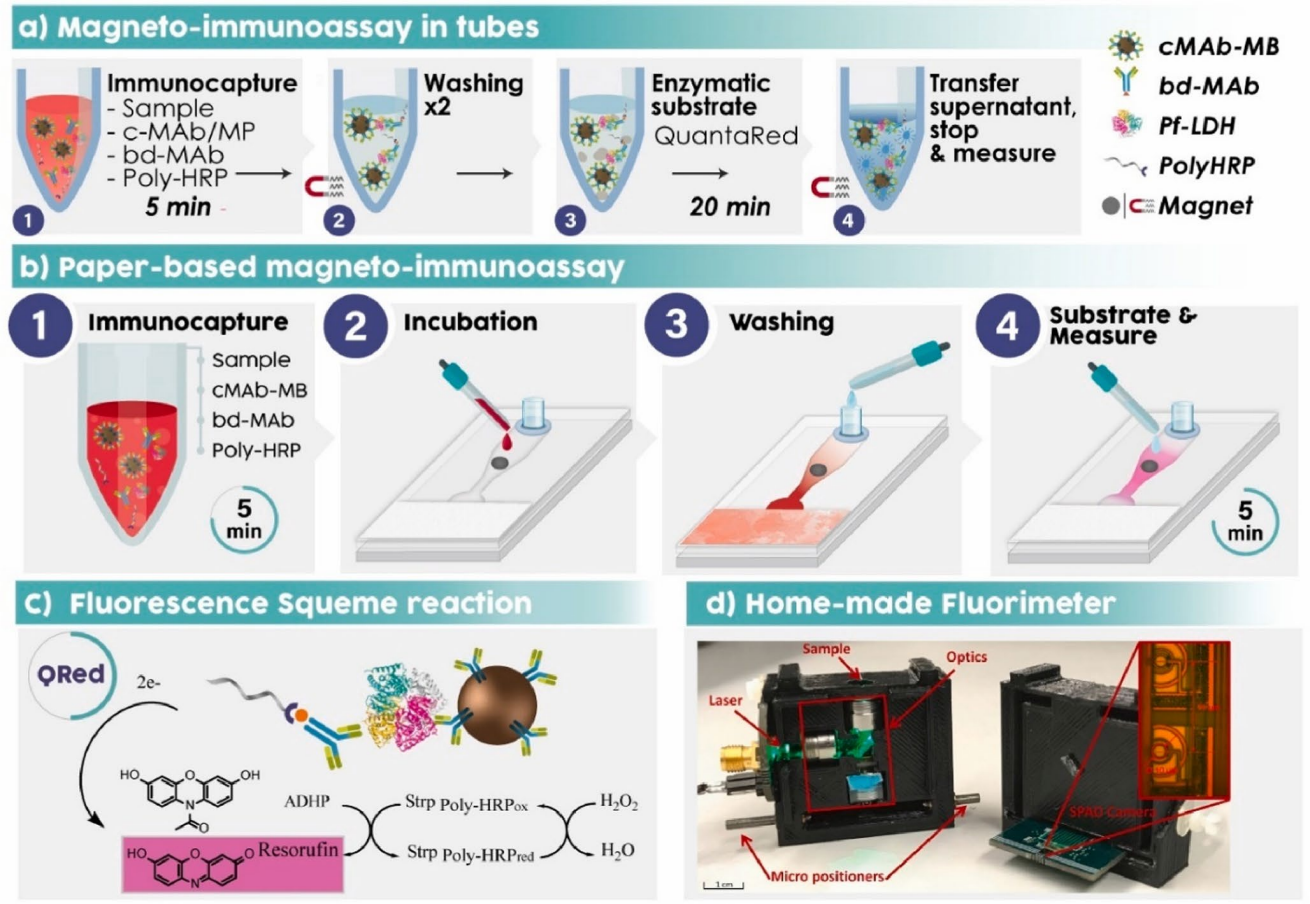
Schematic representation of the single-step magneto-immunoassay (**a**) and the partial paper-based automation (**b**), in which magnetic particle washing, concentration, and fluorescent detection (**c**) The reaction catalysed by Poly-HRP on QuantaRed substrate solution. (**d**) Portable fluorimeter. Reprinted from Ref. [65] with permission from Elsevier

A study by Arias-Alpizar and coworkers introduces a magneto-immunoassay-based POCT device for rapid, sensitive detection of PfLDH (Fig. 7) [65]. The device includes a single-piece paper chip cut with a low-cost craft cutter, placed on a plastic base with a magnet, and completed with a solution reservoir and an adsorption pad. This is used to automate a rapid magneto-immunoassay, in which whole blood is lysed for 5 min to release the antigen, then incubated for 5 more min with a cocktail of reagents. The mixture is subsequently applied to the paper device for on-chip washing and fluorescence detection using a portable, customized reader. With a detection limit of 0.92 ng/mL, this device provides quantitative results in 20 min and is more sensitive than commercial rapid diagnostic tests. This offers a low-cost, portable, and user-friendly solution for malaria diagnosis.

A similar approach was exploited by this team to develop a colorimetric microfluidic paper-based device (µPAD) for the detection of PfLDH, which can be stored at room temperature and manufactured at low cost [66]. This system processes lysed blood samples with a one-step 5-min magnetic immunoassay using magnetic beads, detection antibodies, and a Poly-HRP enzymatic signal amplifier, followed by on-chip washing and detection with a chromogenic substrate solution. The colorimetric signal is measured and analyzed through visual inspection (semi-quantitative) or a smartphone camera (quantitative). Quantitative PfLDH detection is achieved in 15 min with a LOD of 1.4 ng/mL. Tests on clinical samples show that the results of this device are comparable to those of an ELISA and more sensitive than commercial rapid diagnostic tests. These findings suggest that µPAD-based magneto-immunoassays can be adapted for POC testing and that partial automation is feasible.

A number of works have described SPR-based biosensors for the detection of malaria biomarkers. For example, Kamani et al. introduced a novel ohm-shaped refractive index biosensor for the early detection of *Plasmodium*-infected RBCs [69]. The sensor demonstrated high sensitivity and accuracy, with an LOD as low as 0.0007 refractive index units (RIU) for the schizont stage of the parasite. In a different approach, researchers developed an SPR biosensor incorporating zinc oxide, ferric oxide, and black phosphorus [71]. This sensor effectively detected malaria at various stages, with a refractive index sensing range of 1.369–1.409 and high angular sensitivity, particularly during the ring stage of the parasite lifecycle. Another study presented a microstructure optical fiber sensor based on SPR with an open-loop analyte channel for malaria parasite detection [72]. Using a curved surface structure to enhance coupling, the sensor achieved a maximum wavelength sensitivity of 257.8 μm/RIU over a refractive index range of 1.33 to 1.43. To improve detection, a photonic crystal fiber sensor based on SPR was reported, featuring a dual-square-groove design that positioned the plasmonic layer closer to the core [73]. This configuration enhanced sensitivity, facilitating effective malaria diagnosis.

Another study proposed an ultra-sensitive bimetallic SPR biosensor for detecting malaria at various stages [74]. The device uses a Kretschmann configuration with layers of BK7 prism, silver (Ag), zinc telluride (ZnTe), and lead titanate (PbTiO3). It employs angular interrogation for detection and covers a refractive index (RI) range from 1.370 to 1.410, achieving an optimized angular sensitivity of 253°/RIU. The sensor can detect different parasite cycle stages (ring, trophozoite, and schizont) with refractive indices of 1.396, 1.381, and 1.371, respectively, and shows high angular sensitivities of 364°/RIU, 296°/RIU, and 267°/RIU. The SPR sensor can also detect cancerous cells. MATLAB 2017b was used for simulation.

These advancements in optical biosensors underscore their potential to significantly enhance malaria diagnostics by providing rapid, sensitive, and specific detection capabilities.

### Piezoelectric biosensors

Piezoelectric biosensors are an emerging technology with significant potential for detecting disease biomarkers. These biosensors utilize the piezoelectric effect, where mechanical stress or strain applied to a piezoelectric material generates an electrical charge. When a biomarker binds to a bioreceptor on the transducer, it alters parameters such as resonant frequency, mass, or viscosity, thereby altering the electrical output, which can be measured and analyzed. The three types of piezoelectric sensors applied more often to the development of biosensors are the quartz crystal microbalance (QCM), cantilevers, and surface acoustic wave devices. Quartz crystal microbalance (QCM)-based systems enable direct mass-sensitive detection without labeling. Nevertheless, environmental vibrations, temperature fluctuations, and long-term surface functionalization stability remain practical challenges for decentralized deployment. Future developments should prioritize miniaturization, environmental stabilization, and integration with portable electronics to enhance real-world applicability. Recent research has highlighted the promise of piezoelectric biosensors in detecting malaria. For instance, sensors have been developed to detect PfHRP2 and pLDH with high sensitivity and specificity. These sensors have shown promise in laboratory settings, with efforts underway to adapt them for field use [84–88].

**Fig. 8:**
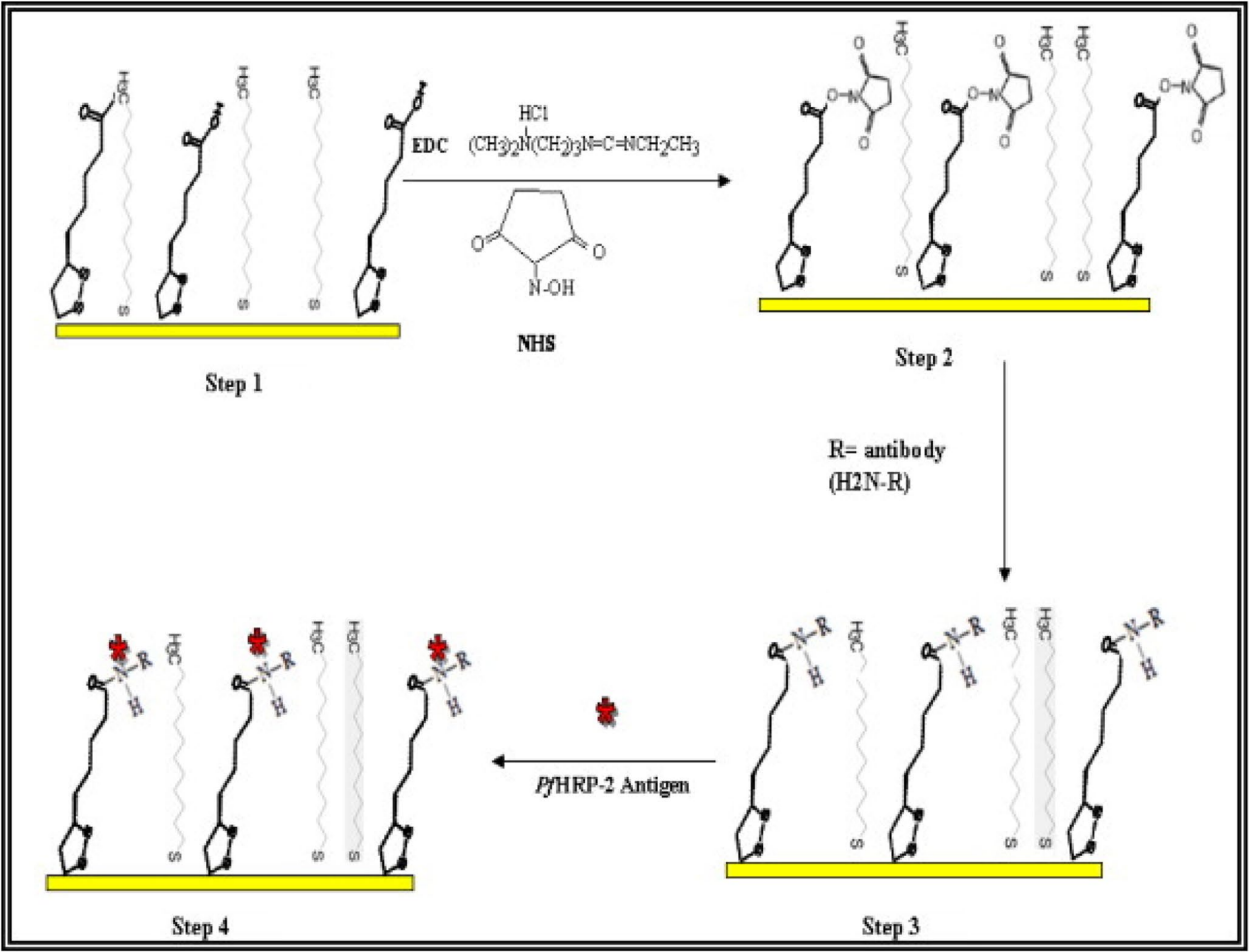
The schematic diagram for piezoelectric immunosensor preparation. Reprinted from Ref. [84] with permission from Elsevier

A new piezoelectric immunosensor was created by Sharma et al. to directly detect the PfHRP2 antigen (Fig. 8) [84]. Mixed SAMs of 1-dodecanethiol and thioctic acid were created on the Au surface of a quartz crystal. The NHS/EDC activation approach was used to link the rabbit anti-PfHRP2 antibodies on the quartz crystal’s mixed SAM-modified gold surface. With a 12 ng/ml LOD, the immunosensor was used to identify PfHRP2 i within the 15–60 ng/mL linear response range. Additionally, 50% of the activity persisted even after 14 days of storage. This technique was used to test clinical human serum samples, and the outcomes matched those from an RDT kit that was sold commercially (NOW^®^ Malaria). In addition to direct antigen detection, piezoelectric MEMS-based sensors have been developed to enhance diagnostic capabilities. For instance, a MEMS sensor utilizing a poly-silicon cantilever structure demonstrated the potential to detect multiple tropical diseases, including malaria, with impressive accuracy [16]. This sensor detects mechanical fluctuations through electromagnetic induction, helping to measure fluctuations in the cantilever’s resonant frequencies, crucial for identifying disease-causing particles. The sensor’s performance has been tested with various pathogens, showing promising results: the voltages generated by the biosensor for Zika, chikungunya, dengue, and malaria pathogens were 1.98 × 10^− 4^, 2.04 × 10^− 4^, 2.23 × 10^− 4^, and 3.43 × 10^− 4^, respectively. These results demonstrate the sensor’s potential for simultaneously accurately detecting multiple tropical and subtropical diseases.

## Conclusions and perspectives

POC diagnostic technologies are key in diagnosing and controlling malaria and are becoming essential in the global fight against this disease, especially in resource-constrained environments. As technology evolved in its transition from the centralized laboratory to the field, users demanded rapid response, sensitivity, accuracy, and ease of use. Healthcare professionals expect to detect Plasmodium infection early to treat patients as soon as possible and avoid complications. However, global malaria management requires more ambitious objectives. The identification of asymptomatic carriers, who display extremely low parasite loads but can still transmit the disease, is crucial to controlling the spread of malaria. Determining the parasite species is important for guiding drug selection. Parasite quantification allows stratifying patients according to disease severity, deciding the therapeutic path, studying patient response to treatment, and anticipating the evolution of drug resistance. Accordingly, an ideal POCT device should provide ultrasensitive, quantitative, and multiplexed detection of the different species of Plasmodium that infect humans, entail minimal user handling, and deliver results in less than 20 min. The analysis should also be carried out in small volumes of sample (1–50 µL, optimally 1–10 µL), obtained non-invasively or minimally invasively (such as saliva or peripheral whole blood from finger stick or heel prick in infants). And the device should enclose all the needed reagents and positive/negative controls, be stable for months at room temperature, portable, and operated without the need for additional equipment or special facilities.

RDTs are widely used in the field for this purpose, displaying numerous advantages that have changed how malaria is diagnosed, treated, and prevented. Compared to microscopy, RDTs are easier and faster to use, and can be employed even by non-specialized trained personnel without sophisticated equipment or facilities. Advances have been made to provide objectivity in RDT readouts using handheld readers, which can also yield semi-quantitative results. Nevertheless, even the best-performing RDTs exhibit limitations in detection limit, quantification, and multiplexing, as well as reproducibility between devices and production batches, which makes them poor competitors with molecular diagnostic methods such as PCR. In turn, molecular methods are the most sensitive technology available for malaria diagnosis, are (semi)quantitative, and are compatible with assay multiplexing. However, they are costly and rely on well-trained personnel and sophisticated equipment and installations, which impedes their broad implementation as POCT technology.

In this context, POC biosensors stand out as versatile and cost-efficient alternatives well-suited to play a role in global malaria control. Different transduction techniques have been employed to produce biosensors for the detection of a range of malaria biomarkers. Recent developments in nanotechnology, materials science, and biotechnology are combining to lay the groundwork for the generation of fast, sensitive, and quantitative responses needed for malaria POC diagnosis. The integration of mobile detection technology, wireless connectivity, or artificial intelligence training is envisioned to enhance the portability and connectivity of POC biosensors, facilitating real-time diagnostics even in remote areas. As the technology continues to improve and integrate with electronic health systems, this will facilitate better control and, eventually, elimination of malaria through effective programs. Nevertheless, additional work will be needed to overcome the existing challenges. Efforts will have to be directed towards improving the sensitivity and selectivity of the existing biosensors, prioritizing those technologies compatible with multiplexed detection, and making them more widely available and affordable for mass production and use. In this context, architectures that are easily adaptable for the detection of additional or alternative analytes, such as other infectious agents, may play a leading role. A good example is the magneto-immunosensors, in which generic transducers can be used to detect alternatively different assays carried out on magnetic beads.

Future next-generation POCT biosensors should prioritize multiplex capability (e.g., simultaneous detection of PfHRP2, pLDH, and PfGDH), ultrasensitive detection of submicroscopic parasitemia, and fully integrated sample-to-answer automation. Integration with wireless connectivity, cloud-based epidemiological surveillance systems, and AI-assisted result interpretation may further enhance outbreak monitoring and disease control efforts.

It can be concluded that next-generation POCT biosensors should be flexible and cost-effective to play a role in the fight against malaria, especially in areas with high malaria incidence and limited access to health facilities. Only in this way can implementing the POC biosensing technology in the field affect the fight against malaria, enhance the effectiveness of health care delivery and prevention programs, and become a cornerstone in the global effort to eradicate malaria.

## Data Availability

No datasets were generated or analysed during the current study.

## Declarations

### Clinical trial number

Clinical trial number: Not applicable.
